# Predictors and outcomes of *Mycobacterium tuberculosis* bacteremia among patients with HIV and tuberculosis co-infection enrolled in the ACTG A5221 STRIDE study

**DOI:** 10.1186/s12879-014-0735-5

**Published:** 2015-01-13

**Authors:** John A Crump, Xingye Wu, Michelle A Kendall, Prudence D Ive, Johnstone J Kumwenda, Beatriz Grinsztejn, Ute Jentsch, Susan Swindells

**Affiliations:** Department of Medicine, Division of Infectious Diseases and International Health, Durham, NC 27710 USA; Department of Pathology, Duke University Medical Center, Box 102359, Durham, NC 27710 USA; Duke Global Health Institute, Duke University, Box 90519, Durham, NC 27708 USA; Kilimanjaro Christian Medical Centre, PO Box 3010, Moshi, Tanzania; Kilimanjaro Christian Medical University College, Tumaini University, PO Box 2240, Moshi, Tanzania; Center for Biostatistics in AIDS Research, Harvard School of Public Health, 651 Huntington Avenue, Boston, MA 02115 USA; Department of Medicine, University of the Witwatersrand, Private Bag 3, Wits 2050, Johannesburg, South Africa; School of Pathology, University of the Witwatersrand, Private Bag 3, Wits 2050, Johannesburg, South Africa; Department of Internal Medicine, College of Medicine, University of Malawi, Private Bag 360, Chichiri, Blantyre 3, Malawi; Evandro Chagas Clinical Research Institute, Oswaldo Cruz Foundation, Avenue Brasil 4365, Manguinhos 21040-360, Rio de Janeiro, Brazil; Division of Infectious Diseases, Department of Internal Medicine, University of Nebraska Medical Center, 42nd and Emile, Omaha, NE 68198 USA

**Keywords:** Africa, Asia, Bacteremia, HIV, Treatment outcome, Tuberculosis

## Abstract

**Background:**

We evaluated predictors and outcomes of *Mycobacterium tuberculosis* bacteremia among participants undergoing baseline mycobacterial blood culture in the ACTG A5221 STRIDE study, a randomized clinical trial comparing earlier with later ART among HIV-infected patients suspected of having tuberculosis with CD4-positive T-lymphocyte counts (CD4 counts) <250 cells/mm^3^. We conducted a secondary analysis comparing participants with respect to presence or absence of *M. tuberculosis* bacteremia.

**Methods:**

Participants with a baseline mycobacterial blood culture were compared with respect to the presence or absence of *M. tuberculosis* bacteremia. Baseline predictors of *M. tuberculosis* bacteremia were identified and participant outcomes were compared by mycobacteremia status.

**Results:**

Of 90 participants with baseline mycobacterial blood cultures, 29 (32.2%) were female, the median (IQR) age was 37 (31–45) years, CD4 count was 81 (33–131) cells/mm^3^, HIV-1 RNA level was 5.39 (4.96–5.83) log_10_ copies/mL, and 18 (20.0%) had blood cultures positive for *M. tuberculosis*. In multivariable analysis, lower CD4 count (OR 0.85 per 10-cell increase, p = 0.012), hemoglobin ≤8.5 g/dL (OR 5.8, p = 0.049), and confirmed tuberculosis (OR 17.4, p = 0.001) were associated with *M. tuberculosis* bacteremia. There were no significant differences in survival and AIDS-free survival, occurrence of tuberculosis immune reconstitution inflammatory syndrome (IRIS), or treatment interruption or discontinuation by *M. tuberculosis* bacteremia status. IRIS did not differ significantly between groups despite trends toward more virologic suppression and greater CD4 count increases at week 48 in the bacteremic group.

**Conclusions:**

Among HIV-infected tuberculosis suspects, lower CD4 count, hemoglobin ≤8.5 g/dL, and the presence of microbiologically confirmed pulmonary tuberculosis were associated with increased adjusted odds of mycobacteremia. No evidence of an association between *M. tuberculosis* bacteremia and the increased risk of IRIS was detected.

**Trial registration:**

ClinicalTrials.gov: NCT00108862.

## Background

Disseminated tuberculosis is strictly defined as isolation of *Mycobacterium tuberculosis* from blood or bone marrow, liver biopsy, or from specimens from ≥2 noncontiguous organs [1]. Disseminated tuberculosis is associated with compromised cell-mediated immunity and the bacteremic form is rapidly fatal in a large proportion of HIV-infected patients [2]. Early recognition and treatment are likely to be important in improving patient outcomes. Although research has identified a number of clinical and laboratory features that may assist with recognition of patients with bacteremic disseminated tuberculosis [2,3], non-specific clinical findings and lack of typical features of pulmonary tuberculosis complicate diagnosis. Randomized trials have confirmed that early initiation of antiretroviral therapy (ART) is associated with improved outcomes for HIV and tuberculosis co-infected patients [4-7]. However, these studies have focused primarily on pulmonary tuberculosis, leaving unanswered questions about the timing and impact of ART in patients with confirmed disseminated tuberculosis. Research suggests that patients with disseminated and extra-pulmonary forms of tuberculosis may represent a special group that may experience worse outcomes, including greater risk for the tuberculosis immune reconstitution inflammatory syndrome (IRIS) [8,9].

In order to strengthen data on clinical predictors of *M. tuberculosis* bacteremia and to assess the effect of *M. tuberculosis* bacteremia on ART treatment outcomes and toxicities, we conducted a planned analysis of patients enrolled in the AIDS Clinical Trials Group (ACTG) A5221 strategy study of early versus later initiation of antiretroviral therapy for HIV-infected persons treated for tuberculosis with CD4 < 250 cells/mm^3^ (STRIDE), who received a mycobacterial blood culture as part of their baseline evaluation. Participants with and without bacteremic disseminated tuberculosis were compared.

## Methods

### ACTG A5221 STRIDE study

A5221 was an open-label, randomized study comparing earlier ART (within 2 weeks after initiation of treatment for tuberculosis) with later ART (between 8 and 12 weeks after initiation of treatment for tuberculosis) in HIV-1 infected patients with CD4-positive T-lymphocyte counts (CD4 counts) <250 cells/mm^3^ and suspected tuberculosis. The primary endpoint was the proportion of patients who survived without a new (previously undiagnosed) acquired immunodeficiency syndrome (AIDS)-defining illness at 48 weeks. Mycobacterial blood cultures were not required by the protocol. However, all tuberculosis diagnostic information was required to be recorded by the study sites. Mycobacterial blood cultures were not standardized across sites, but were collected at the discretion of the study team prior to the administration of tuberculosis treatment and antiretroviral therapy, and were processed in laboratories adhering to Good Clinical Laboratory Practice standards. Confirmed tuberculosis was defined as detection of acid-fast bacilli in sputum smear or lymph node specimen, or a positive culture for *Mycobacterium tuberculosis* from sputum, lymph node, or another sterile site. Probable tuberculosis required clinician’s assessment that signs and symptoms warranted empiric tuberculosis treatment. Detailed methods and results of the study are published elsewhere [7,10] and available at www.clinicaltrials.gov identifier NCT00108862. The quality of the data was assured by adherence to Good Clinical Practice and Good Clinical Laboratory Practice standards.

### Mycobacteremia sub-study

Of 806 eligible participants from 26 sites enrolled in A5221, 90 (11.2%) participants from 5 (19.2%) sites had baseline mycobacterial blood cultures and were included in this analysis.

### Statistical analysis

Exact logistic regression models were used to investigate baseline predictors of *M. tuberculosis* bacteremia. Covariates with p ≤ 0.10 from an exact conditional score test in univariable models were examined together in a multivariable model using the stepwise selection method. Estimated proportions of participants who survived with or without a new AIDS event at 48 weeks and failure-time plots were calculated with the use of the Kaplan–Meier method. Failure was defined at the first qualifying event. Failure times were determined as the differences between randomization and failure dates or, if censored, last clinical visit dates.

An asymptotic normal two-sample test was employed to evaluate the difference in failure proportions. Fisher’s exact and exact Wilcoxon tests were used to assess between-group differences. Based on the sample sizes and numbers of primary endpoint, there was 12% power to detect a difference using a two-sided 0.05-level binomial test.

Participant characteristics at baseline, including sex; enrollment at an African site; age; CD4 count; HIV-1 RNA level; presence of an AIDS-defining illness; body mass index (BMI); hemoglobin; self-reported cough, weight loss, and fever; and confirmed tuberculosis, were evaluated as potential predictors of *M. tuberculosis* bacteremia in a univariable exact logistic regression model.

### Research ethics

The protocol was approved by the following institutional review boards or ethics committees: New York University School of Medicine, NY, USA; Los Angeles Biomedical Research Institute, CA, USA; University of California San Diego, CA, USA; University of California San Francisco, CA, USA: University of Southern California, CA, USA; Lifespan Miriam Hospital, RI, USA; University of the Witwatersrand, Johannesburg, South Africa; University of KwaZulu Natal, Durban, South Africa; Asociacion Civil Impacta Salud y Educacion, Lima, Peru; Chiang Mai University Research Institute for Health Sciences and Faculty of Medicine Chiang Mai University, Chiang Mai, Thailand; National AIDS Research Institute, Pune, India; YRG Care Medical Centre, Chennai, India; National Health Science Research Committee, Lilongwe, Malawi, and University of North Carolina, NC, USA; Instituto de Pequisa Clinica Evandro Chagas, Rio de Janeiro, Brazil; Grupo Hospitalar Conceicao, Porto Alegre, Brazil; Joint Clinical Research Centre and Uganda National Council for Science and Technology, Kampala, Uganda; Kenya Medical Research Institute, Nairobi, Kenya; Moi University College of Health Sciences, Eldoret, Kenya; Health Research and Knowledge Management, Gaborone, Botswana, and Harvard School of Public Health, MA, USA; University of Zambia, Lusaka, Zambia, and University of Alabama at Birmingham, AL, USA; Comite des Droits Humains des Centres GHESKIO, Port-au-Prince, Haïti; Johns Hopkins University, MD, USA, and University of Malawi, Blantyre, Malawi; Medical Research Council of Zimbabwe, Harare, Zimbabwe; Hospital Universitario Clementino Fraga Filho, Rio de Janeiro, Brazil; Bronx-Lebanon Hospital Center, NY, USA; University of Texas Health Science Center at Houston, TX, USA; and University of Medicine and Dentistry of New Jersey Newark Campus, NJ, USA. All participants provided written informed consent.

## Results

### Participant characteristics

Eighty-eight (97.8%) of 90 participants with baseline mycobacterial blood cultures were from sites in Rio de Janeiro, Brazil; Lilongwe, Malawi; and Johannesburg, South Africa. Among all participants from these 3 sites, those with mycobacterial blood cultures were more often enrolled at a non-African site (p < 0.001), older (p = 0.017), and more likely to report cough (p = 0.022) and weight loss (p < 0.001) compared with participants without cultures (Table 1). Of all 90 participants with baseline mycobacterial blood cultures, 29 (32.2%) were female, the median (IQR) age was 37 (31–45) years, CD4 count was 81 (33–131) cells/mm^3^, and HIV-1 RNA level was 5.39 (4.96–5.83) log_10_ copies/mL. Forty-two (46.7%) of 90 participants started early ART and 18 (20.0%) of 90 were blood culture positive for *M. tuberculosis*. Of those with mycobacteremia, 10 (55.6%) had positive baseline sputa for acid-fast bacilli, and 17 (94.4%) met the study definition of confirmed tuberculosis and 1 (5.6%) met the definition of probable tuberculosis at week 12.

**Table 1:** **Baseline characteristics by blood culture availability among participants enrolled at the three sites that collected nearly all the baseline blood cultures, ACTG A5221 STRIDE study**

	**Baseline blood culture available**									
	**Total**			**Yes**			**No**			**p-value** ^**a**^
	**n/**	**n**	**(%)**	**n/**	**n**	**(%)**	**n/**	**n**	**(%)**	
**Baseline characteristics**										
Male sex	137/	226	(60.6)	59/	88	(67.0)	78/	138	(56.5)	0.126
Enrollment at an African site	158/	226	(69.9)	38/	88	(43.2)	120/	138	(87.0)	<0.001
Age (years)^b^	34.0 (29.0, 42.0)			36.5 (30.5, 44.5)			33.0 (28.0, 41.0)			0.017
CD4 count (cells/mm^3^)^b^	81.5 (37.0, 132.0)			81.5 (31.5, 131.5)			79.5 (41.0, 134.0)			0.820
HIV RNA (log_10_ copies/mL)^b^	5.4 (5.0, 5.8)			5.4 (5.0, 5.8)			5.4 (5.0, 5.8)			0.796
Presence of an AIDS-defining illness	16/	226	(7.1)	8/	88	(9.1)	8/	138	(5.8)	0.427
Body mass index (BMI, kg/m^2^)^b^	19.2 (17.5, 21.5)			18.9 (17.2, 20.9)			19.3 (17.8, 21.9)			0.236
Hemoglobin ≤8.5 g/dL	43/	226	(19.0)	17/	88	(19.3)	26/	138	(18.8)	1.000
Self-reported cough	4/	226	(1.8)	4/	88	(4.5)	0/	138	(0.0)	0.022
Self-reported weight loss	11/	226	(4.9)	11/	88	(12.5)	0/	138	(0.0)	<0.001
Self-reported fever	2/	226	(0.9)	2/	88	(2.3)	0/	138	(0.0)	0.151
Confirmed tuberculosis at entry	111/	226	(49.1)	48/	88	(54.5)	63/	138	(45.7)	0.220

^a^Fisher’s Exact test, except for age, CD4 count, HIV RNA, and BMI which were exact Wilcoxon test.

^b^Median (IQR) values are presented for age, CD4 count, HIV RNA, and BMI.

### Baseline predictors of *M. tuberculosis* bacteremia

The covariates that were significantly (p ≤ 0.10) associated with higher odds of *M. tuberculosis* bacteremia were female sex (OR 2.6, 90% CI 0.93, 7.2; p = 0.092), younger age (OR 0.94, 90% CI 0.89, 0.99; p = 0.058), lower CD4 count (OR 1.2 per 10-cell decrease, 90% CI 1.1, 1.4; p = 0.003), higher HIV-1 RNA level (OR 2.2, 90% CI 1.1, 4.8; p = 0.048), lower BMI (OR 0.82, 90% CI 0.69, 0.96; p = 0.053), hemoglobin ≤8.5 g/dL (OR 5.5, 90% CI 1.8, 17.1; p = 0.005), and confirmed tuberculosis (OR 19.6, 90% CI 3.4, 424.2; p = 0.003). In the multivariable analysis, lower CD4 count (adjusted OR 1.2 per 10-cell decrease, 90% CI 1.0, 1.4; p = 0.012), hemoglobin ≤8.5 g/dL (adjusted OR 5.8, 90% CI 1.2, 34.4; p = 0.049), and confirmed tuberculosis (adjusted OR 17.4, 90% CI 2.6, 410.1; p = 0.001) were associated with increased adjusted odds of *M. tuberculosis* bacteremia.

### Survival and survival without a new AIDS-defining event at 48 weeks

**Figure 1:**
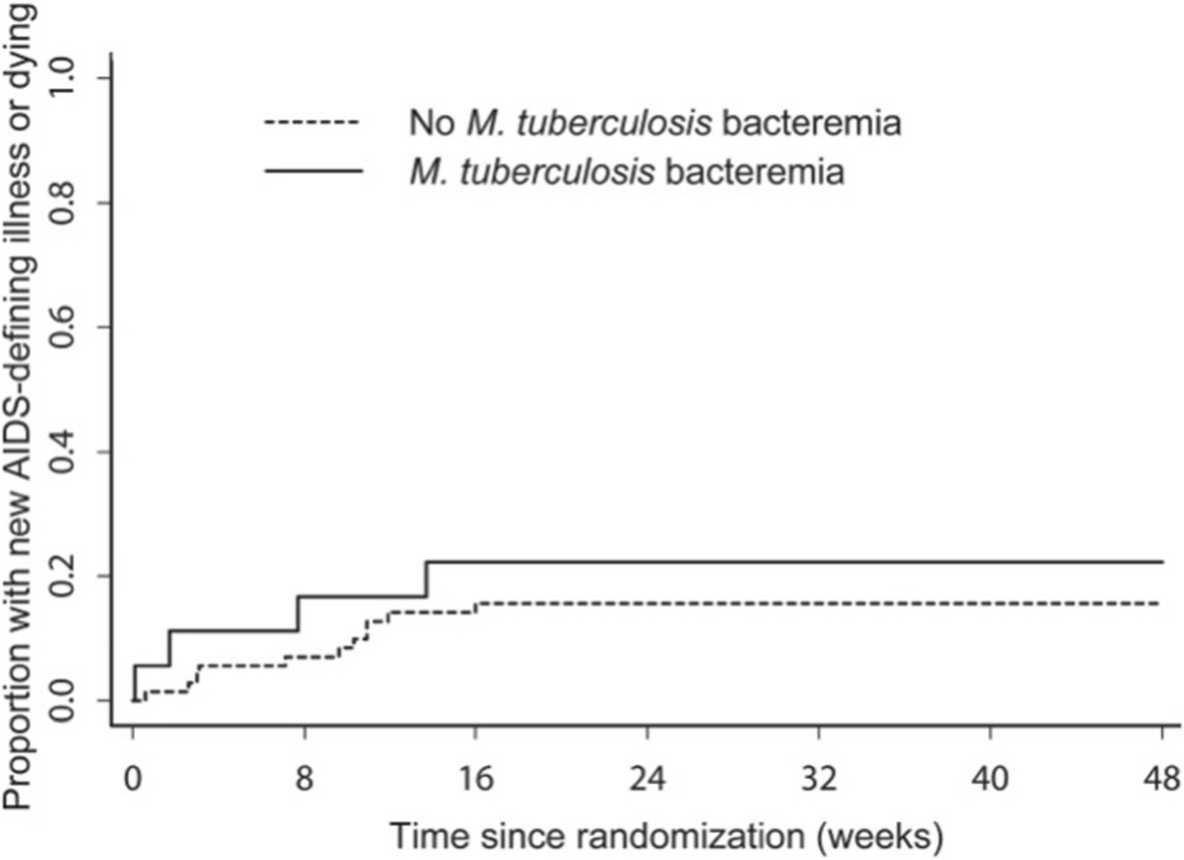
**Time-to-first new AIDS-defining illness or death among participants with and without mycobacteremia, ACTG A5221 STRIDE study.**

There were 2 (11.1%) deaths out of 18 participants in the group with *M. tuberculosis* bacteremia compared with 6 (8.3%) deaths out of 72 participants in the non-bacteremic group. The estimated difference in proportions at week 48 between the two groups was −2.5% (95% CI −18.5%, 13.4%; p = 0.76). There were 4 (22.2%) events (1 death and 3 new AIDS-defining illnesses (1 then died) out of 18 participants in the group with *M. tuberculosis* bacteremia compared with 11 (15.3%) events (4 deaths and 7 new AIDS-defining illnesses (2 then died)) out of 72 participants in the group without. The estimated difference in event proportions at week 48 was −6.6% (95% CI −27.6%, 14.3%; p = 0.54). No significant differences were observed using Fisher’s exact test in subgroup analyses (within CD4 < 50 cells/mm^3^ and ≥50 cells/mm^3^; early ART and late ART). The Figure 1 shows time-to-first new AIDS-defining illness or death.

### Other participant outcomes by *M. tuberculosis* bacteremia status

Table 2 shows participant outcomes by *M. tuberculosis* bacteremia status. There were no differences by *M. tuberculosis* bacteremia in the occurrence of IRIS, in interruption or discontinuation of tuberculosis medications or ART due to drug toxicity, or in sputum culture conversion from positive at baseline to negative at week 8. Participants with *M. tuberculosis* bacteremia were significantly more likely to convert their sputum smear from positive at baseline to negative at week 8 (70% versus 20%; p = 0.008). The higher proportion of participants with sputum conversion in the *M. tuberculosis* bacteremia group remained significant when missing baseline smear results were coded as positive (p = 0.036) or as negative (p = 0.007). The difference remained significant in an analysis adjusting for timing of ART initiation and CD4 count strata (exact logistic regression *M. tuberculosis* bacteremia group conditional score test p = 0.02). There were non-significant trends in participants with *M. tuberculosis* bacteremia being more likely to have HIV-1 RNA levels <400 copies/mL at week 48 (p = 0.062) and experiencing CD4 count increases ≥100 cells/mm^3^ at week 48 (p = 0.059).

**Table 2:** **Participant characteristics and outcomes by** ***M. tuberculosis*** **bacteremia status, ACTG A5221 STRIDE study**

	***M. tuberculosis*** **bacteremia**									
	**Total**			**Yes**			**No**			**p-value** ^**a**^
	**n/**	**n**	**(%)**	**n/**	**n**	**(%)**	**n/**	**n**	**(%)**	
**Participant characteristics**										
Male sex	61/	90	(67.8)	9/	18	(50.0)	52/	72	(72.2)	0.092
Enrollment at an African site	38/	90	(42.2)	9/	18	(50.0)	29/	72	(40.3)	0.595
Age (years)^b^	37.0 (31.0, 45.0)			33.0 (29.0, 37.0)			38.0 (31.0, 45.4)			0.056
CD4 count (cells/mm^3^)^b^	81.0 (33.0, 131.0)			35.0 (16.0, 62.0)			91.0 (39.0, 146.5)			<0.001
HIV RNA (log_10_ copies/mL)^b^	5.4 (5.0, 5.8)			5.7 (5.4, 5.9)			5.3 (4.8, 5.8)			0.019
Presence of an AIDS-defining illness	8/	90	(8.9)	2/	18	(11.1)	6/	72	(8.3)	0.658
Body mass index (BMI, kg/m^2^)^b^	18.9 (17.2, 20.9)			17.3 (15.6, 20.6)			19.2 (17.9, 21.1)			0.026
Hemoglobin ≤8.5 g/dL	17/	90	(18.9)	8/	18	(44.4)	9/	72	(12.5)	0.005
Self-reported cough	4/	90	(4.4)	1/	18	(5.6)	3/	72	(4.2)	1.000
Self-reported weight loss	12/	90	(13.3)	4/	18	(22.2)	8/	72	(11.1)	0.248
Self-reported fever	2/	90	(2.2)	0/	18	(0.0)	2/	72	(2.8)	1.000
Confirmed tuberculosis at entry	50/	90	(55.6)	17/	18	(94.4)	33/	72	(45.8)	<0.001
Early ART initiation	42/	90	(46.7)	9/	18	(50.0)	33/	72	(45.8)	0.796
**Outcome**										
Immune reconstitution inflammatory syndrome	11/	90	(12.2)	2/	18	(11.1)	9/	72	(12.5)	1.000
HIV-1 RNA level <400 copies/mL at week 48	69/	82	(84.1)	16/	16	(100.0)	53/	66	(80.3)	0.062
CD4 count increase ≥100 cells/mm^3^ at week 48	63/	85	(74.1)	15/	16	(93.8)	48/	69	(69.6)	0.059
Interruption or discontinuation of tuberculosis medications	22/	90	(24.4)	7/	18	(38.9)	15/	72	(20.8)	0.131
Interruption or discontinuation of ART^c^	7/	85	(8.2)	3/	16	(18.8)	4/	69	(5.8)	0.120
Sputum smear conversion baseline to week 8^d^	12/	35	(34.3)	7/	10	(70.0)	5/	25	(20.0)	0.008
Sputum culture conversion baseline to week 8^d^	17/	23	(73.9)	3/	4	(75.0)	14/	19	(73.7)	1.000

^a^Fisher’s Exact test, except for age, CD4 count, HIV RNA, BMI, sputum smear and culture conversion which were exact Wilcoxon test.

^b^Median (IQR) values are presented for age, CD4 count, HIV RNA, and BMI.

^c^ART: antiretroviral therapy.

^d^From positive to negative.

## Discussion

We demonstrated that lower CD4 count, hemoglobin ≤8.5 g/dL, and the presence of microbiologically confirmed tuberculosis were associated with increased adjusted odds of *M. tuberculosis* bacteremia among HIV-infected patients suspected to have tuberculosis. We found no significant differences in survival or AIDS-free survival, occurrence of IRIS, or treatment interruption or discontinuation by *M. tuberculosis* bacteremia status. Occurrence of IRIS did not significantly differ between groups [8], despite trends toward more virologic suppression and greater CD4 count increases at week 48 in the group with *M. tuberculosis* bacteremia. Compared with patients without *M. tuberculosis* bacteremia, mycobacteremic patients were more likely to convert their sputum smear from positive at baseline to negative at week 8.

Lower CD4 count, hemoglobin ≤8.5 g/dL, and the presence of microbiologically confirmed tuberculosis may assist clinicians in identifying tuberculosis suspects with *M. tuberculosis* bacteremia and we suggest that these be included in larger studies developing clinical diagnostic strategies for disseminated tuberculosis. Unlike studies of disseminated tuberculosis done among febrile patients, we did not detect an association between cough, fever, or weight loss and *M. tuberculosis* bacteremia. This is probably because A5221 enrolled tuberculosis suspects who would be expected to have cough, fever, or weight loss irrespective of the co-occurrence of *M. tuberculosis* bacteremia.

The case fatality ratio of patients with *M. tuberculosis* bacteremia was much lower in A5221 than in other studies [2,11], and there were no significant differences in survival or AIDS-free survival of HIV and tuberculosis co-infected patients by mycobacteremia status. Possible explanations for the better outcomes observed in this study include that A5221 entry criteria would have excluded the most severely ill patients; initiation of ART and tuberculosis treatment may have been more timely in the clinical trial than would be the case under program conditions; and that some patients with occult presentations of disseminated tuberculosis could have been overlooked through a focus on features of clinical tuberculosis. The lack of differences in survival and AIDS-free survival between the groups may suggest that mycobacteremia does not have the prognostic value in the HIV-infected tuberculosis suspect population that it does in the febrile illness population [12], or may simply be due to lack of power to detect a difference.

The higher total body mycobacterial load assumed to be present in patients with the bacteremic disseminated form of tuberculosis combined with rapid ART-associated immune reconstitution has been suggested to increase the risk for IRIS [13]. It is reassuring that *M. tuberculosis* bacteremia was not associated with increased risk for IRIS in our analysis despite the high proportion achieving virologic suppression and CD4 count increases ≥100 cells/mm^3^ at week 48.

Compared with patients without *M. tuberculosis* bacteremia, mycobacteremic patients were more likely to convert their sputum smear from positive at baseline to negative at week 8. This could reflect differences in mycobacterial burden in the respiratory tract of patients with disseminated tuberculosis compared with those with primarily pulmonary disease.

This analysis had a number of limitations. A5221 was not powered to examine the effect of *M. tuberculosis* bacteremia on patient outcomes. The use of mycobacterial blood culture during the baseline evaluation of ACTG protocol A5221 varied considerably by site and was at the discretion of the site team. This could have introduced bias. Misclassification may have occurred in the control group since not all patients without mycobacteremia were ultimately confirmed to have tuberculosis and a single mycobacterial blood culture is not 100% sensitive for the detection of mycobacteremia.

## Conclusions

Among HIV-infected tuberculosis suspects, mycobacteremia was associated with lower CD4 count, hemoglobin ≤8.5 g/dL, and the presence of microbiologically confirmed tuberculosis. Tuberculosis suspects with mycobacteremia were more likely to convert their sputum smear from positive at baseline to negative at week 8, but did not otherwise significantly differ by outcome compared to those without *M. tuberculosis* bacteremia. Most notably, *M. tuberculosis* bacteremia did not appear to be associated with increased risk for IRIS despite the high proportion achieving virologic suppression and CD4 count increases ≥100 cells/mm^3^ at week 48. However, larger studies with standardized and consistent use of mycobacterial blood culture are needed to confirm the findings of our analysis.
